# *In silico* testing to identify compounds that inhibit ClfA and ClfB binding to the host for the formulation of future drugs against *Staphylococcus aureus* colonization and infection

**DOI:** 10.3389/fcimb.2024.1422500

**Published:** 2024-10-01

**Authors:** Shila Kumari Singh, Minakshi Bhattacharjee, Balagopalan Unni, Rajpal Singh Kashyap, Abdul Malik, Suhail Akhtar, Sabiha Fatima

**Affiliations:** ^1^ Faculty of Sciences, Assam Downtown University, Guwahati, Assam, India; ^2^ Department of Research, Central India Institute of Medical Science, Nagpur, Maharasthra, India; ^3^ College of Pharmacy, King Saud University, Riyadh, Saudi Arabia; ^4^ Department of Biochemistry, Andrew Taylor Still University of Health Science, Kirksville, MO, United States; ^5^ College of Applied Medical Sciences, King Saud University, Riyadh, Saudi Arabia

**Keywords:** *Staphylococcus aureus*, clumping factor, adhesin binding protein, antimicrobial resistance (AMR), colonization, molecular docking & molecular dynamics (MD) simulation

## Abstract

**Introduction:**

*Staphylococcus aureus* is a highly resistant pathogen. It has multiple virulence factors, which makes it one of the most pathogenic bacteria for humankind. The vast increase in antibiotic resistance in these bacteria is a warning of existing healthcare policies. Most of the available antibiotics are ineffective due to resistance; this situation requires the development of drugs that target specific proteins and are not susceptible to resistance.

**Methods:**

In this study, we identified a compound that acts as an antagonist of ClfA and ClfB by inhibiting their binding to host cells.

**Results:**

The shortlisted compound’s binding activity was tested by docking and molecular dynamics during its interaction with proteins. The identified compound has excellent binding energy with both ClfA (-10.11 kcal/mol) and ClfB (-11.11 kcal/mol).

**Discussion:**

The molecular dynamics of the protein and compound were stable and promising for further *in vitro* and *in vivo* tests. The performance of our compound was tested and compared with that of the control molecule allantodapsone, which was reported in a previous study as a pan inhibitor of the clumping factor. An ADMET study of our selected compound revealed its reliable drug likeliness. This compound is an ideal candidate for *in vitro* studies.

## 1 Introduction

*Staphylococcus aureus* infections are not new to the world; they are responsible for high death rates due to hospital-acquired infections. Patients admitted to intensive care units, patients with implants, and patients in the postsurgical unit are among the major groups affected by virulent strains of *Staphylococcus aureus* or methicillin-resistant *Staphylococcus aureus* (MRSA) (Foster, 2004; Sampedro and Bubeck Wardenburg, 2017; Troeman et al., 2023). The prevalence of diseases caused by MRSA is not confined to hospitals; it is a major pathogen isolated from community-acquired infections (Berman et al., 1993; DeLeo et al., 2010). It is associated with suppurative infections, skin and soft tissue infection, pneumonia, endocarditis, food poisoning, surgical site infection, etc (Lowy, 1998; Foster, 2004; Tong et al., 2015).

The gradual increase in antibiotic resistance in *Staphylococcus aureus* is a point of concern to the existing healthcare system across the world. It contributes to the pathogenicity and virulence of this bacterium (Otto, 2010). It is considered the most common pathogen with drug resistance and a very high mortality rate following nosocomial infections (Jarvis et al., 2007; Klein et al., 2007; Klevens et al., 2008). The increasing rates of drug resistance development have an impact on the global burden of infections with high severity. This may lead to the exhaustion of antibiotic options for treating this pathogen. Currently, there is a demand for an alternative therapy for most pathogenic strains of *S. aureus* that are not susceptible to existing antibiotics due to drug resistance (Sampedro and Bubeck Wardenburg, 2017; Prencipe et al., 2022). In recent reports, due to the lack of many drug options on the market, newer drugs were under clinical trials; however, the cost for development and testing of only 59 drugs during 2015-2016 was estimated to approximately 19 million dollars, which was a high cost for conventional drugs which bacteria may turn resistant to in future (Moore et al., 2018).

The adhesin proteins present on the surface of Staphylococcus play a significant role in the adherence of the pathogen to host cells. This process is mediated through a receptor on the surface of host cells, also known as a ligand. These proteins belong to a family or group of proteins called MSCRAMM (microbial surface components recognizing adhesive matrix molecules) that represent the surface component of the bacterial cell. Surface adhesin proteins participate in colonization as well as infection (Patti et al., 1994; Peacock et al., 2002). Some important adhesin proteins of *Staphylococcus aureus* are clumping factor (Clf), biofilm-associated protein (Bap), collagen binding protein (Cna) and fibronectin binding protein (FnBP) (Walsh, 2000; Mulcahy et al., 2012; Soltani et al., 2019). These proteins are associated with several virulence factors and are responsible for the multifaceted virulence of *S. aureus*. The formation of biofilms, bacterial accumulation in plasma, cardiovascular diseases through platelet activation, attack on immune cells, etc., are some of the contributions of surface adhesins to staphylococcal infection (Paharik and Horswill, 2016; Josse et al., 2017; Soltani et al., 2019).

Due to increasing resistance to antibiotics and the challenges in treating *S. aureus* and MRSA, alternative methods to develop drugs need to be explored. In this study, we adopted the principle of structure-based drug discovery to develop an antagonist that interrupts ligand-adhesin binding. We selected the clumping factor adhesins ClfA and ClfB for this study because of their roles in both colonization and infection. By molecular docking, various candidates were selected based on their binding to the active sites of ClfA and ClfB. Later, the interactions and properties between the shortlisted compounds and ClfA and ClfB were analyzed by molecular dynamics. We also studied the drug likeness of the selected compounds.

## 2 Materials and methods

### 2.1 Structure-based drug design

After a thorough review of the mechanism and properties of the clumping factor components ClfA and ClfB, their 3D structures were obtained from a protein bank library (www.rcsb.org) (Hawkins et al., 2012). The structure was further refined and processed by the PyMOL Molecular Graphics System, Version 1.2r3pre, Schrödinger, LLC) and minimized using UCSF Chimera version 1.14 software (23). Next, a standard, allantodapsone, was identified for comparison of the identified compound, its interaction pattern with the protein and its drug likeness. The physiochemical properties of allantodapsone were studied in detail at https://pubchem.ncbi.nlm.nih.gov (Prencipe et al., 2022). Compounds similar to allantodapsone were identified from a bank of approved drugs and antimicrobial agents (Frog V 2.14). In our study, the Aurora FC Antibacterial database and DrugBank were utilized for identifying ligand compounds.

Drug likeliness and toxicity analyses of the selected compounds/ligands were performed using SwissADME (http://www.swissadme.ch/) (Sarkar, 2021), whereas toxicity analysis was performed based on parameters such as hepatotoxicity, carcinogenicity, mutagenicity and cytotoxicity using ProTox-II (http://tox.charite.de/protox_II) (Banerjee et al., 2018).

Molecular docking of the 3D structures of ClfA and ClfB with standard and shortlisted compounds was performed by using Autodock 4.2 (Morris et al., 2009). The active site composition of amino acids was determined in a previous study (Prencipe et al., 2022). During docking, the amino acids at the active site of the adhesin proteins (ClfA and ClfB) were kept rigid, while the ligand molecules were allowed to change positions. The PDBQT files of the proteins and ligands were prepared with AutoDock Tools (v.1.5.6) of the MGL software package. The protein-ligand docking complex was analyzed by the Lamarckian genetic algorithm (LGA) method.

The 2D and 3D structures of the docking complex were studied for interpreting the binding energies. The binding affinity was expressed as the binding score. The 2D and 3D structures were obtained from UCSF Chimera version 1.14 (Pettersen et al., 2004) and Accelrys Discovery Studio Visualizer (Dassault Systèmes BIOVIA 2017).

### 2.2 Molecular dynamic simulation

The shortlisted compound/ligand and the standard allantodapsone were studied further by molecular dynamics simulation within a Linux environment created by the Desmond modules of the Schrodinger (Schrödinger release maestro, 2021-4, 2021). The simple point charge water box solvent model in addition to the OPLS2005 force field was employed for the docking complexes made from protein-ligand interactions (Jorgensen and Tirado-Rives, 1988). To create a physiological environment, Na+ and Cl− ions were added for neutralization, which was followed by the addition of 0.15 M NaCl solution. The system was equilibrated by running NVT (number, volume, temperature) and NPT (number, pressure, temperature) ensembles for 100 ns and 12 ns, respectively (Martyna et al., 1992; Martyna et al., 1994). The simulation interaction diagram was generated by using the Desmond simulation interaction diagram tool of Maestro.

The root mean square deviation (RMSD), root mean square fluctuation (RMSF), and hydrogen bonds were estimated for the validation of the results. All the simulations were run in a set of three cycles for both the selected compound and the standard.

## 3 Result

The basis of structure-based drug design is based on the identification of compounds by docking mechanisms and subsequent study of their molecular interactions via simulation.

### 3.1 Molecular docking to select compounds/ligands that bind to ClfA and ClfB

**Figure 1:**
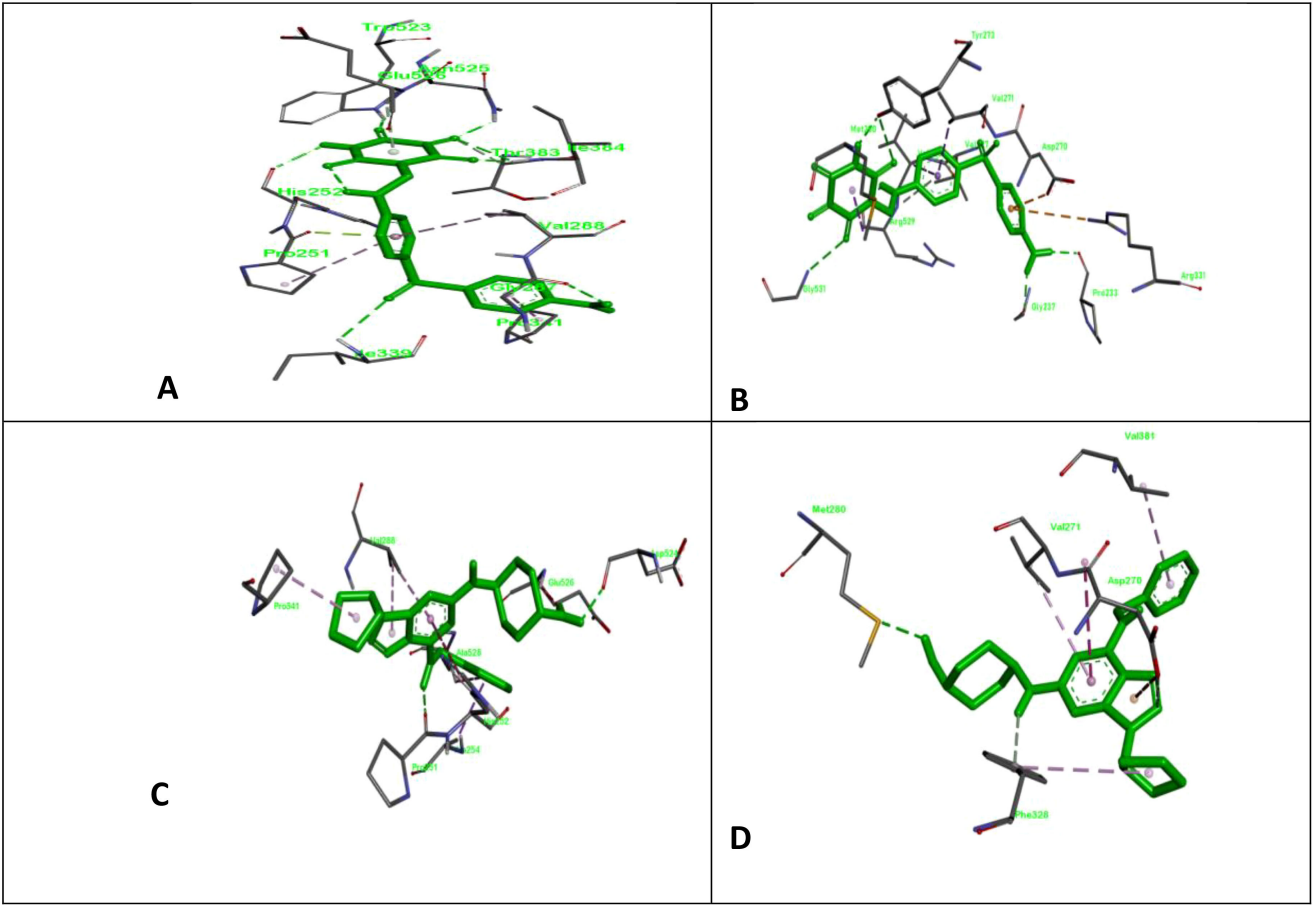
ClfA and ClfB are two components of the clumping factor adhesin protein that participate in ligand adhesin binding. Here, we show the docking complex of ClfA and B with standard and test compounds; **(A)** the docking complex of allantodapsone with ClfA; **(B)** the docking complex of allantodapsone with ClfB; **(C)** the docking complex of 2-[trans-(4-aminocyclohexyl) amino]-6-(benzyl-amino)-9-cyclopentylpurine with ClfA; and **(D)** the docking complex of 2-[trans-(4-aminocyclohexyl) amino]-6-(benzyl-amino)-9-cyclopentylpurine with ClfB.

The docking results revealed a total of 5 compounds with the best binding energies for the ClfA and ClfB proteins. We also evaluated the binding energy of allantodapsone to compare it with that of the standard identified or shortlisted compounds. The binding energy of each shortlisted compound is given in **Table 1** and **Figure 1**.

**Table 1:** Binding energy and other important properties of the shortlisted compounds for ClfA and ClfB.

Sl. No.	Compounds	MW g/mol	H bond acceptor	H- bond donor	Rotatable bonds	Binding energy with ClfA (kcal/mol)	Binding energy with ClfB (kcal/mol)
1	clfa_c_rnase_X-ray_inh3_1jn4_1_26	637.39	17	7	10	-9.6	-9.5
2	clfa_d_rnase_X-ray_inh3_1jn4_1_38	637.39	17	7	10	-9.5	-9.5
3	clfa_c_cdk2_X-ray_inh2_1fvv_1_31	450.51	5	3	5	-9.6	-9.1
4	clfa_d_cdk2_X-ray_inh1_1e9h_1_1	342.33	5	3	1	-9.1	-8.7
5	clfa_d_cdk2_X-ray_inh5_1g5s_1_11	405.54	4	3	6	-10.37	-11.11
6	Allantodapsone ^1^	402.38	6	3	5	-9.25	-9.09

^1^Allantodapsone is used as a standard to compare the performance of shortlisted compounds. As shown in **Table 1**, compared to standard allantodapsone, of the five shortlisted compounds, the ligand cdk2_X-ray_inh5_1g5s_1_11, also known as “2-[trans-(4-aminocyclohexyl) amino]-6-(benzyl-amino)-9-cyclopentylpurine”, had the lowest binding energy, -10.37 and -11.11 kcal/mol for ClfA and ClfB, respectively. In terms of binding energy, cdk2_X-ray_inh5_1g5s_1_11 had better results than allantodapsone. The docking structure of cdk2_X-ray_inh5_1g5s_1_11 and allantodapsone with respect to their binding to ClfA and ClfB is shown in **Figures 1A-D**.

**Figure 2:**
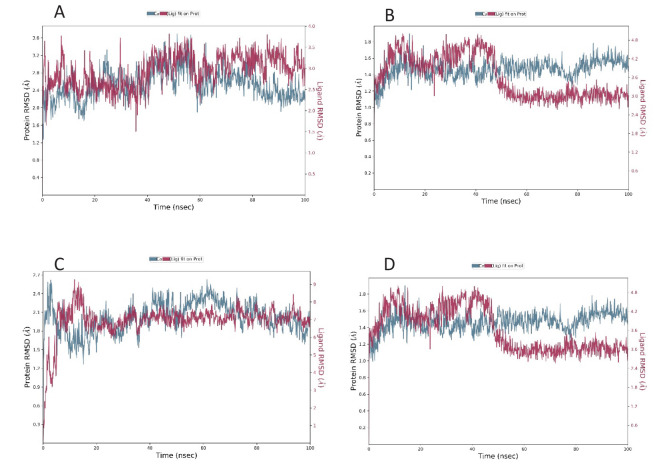
RMSD plot of the docked complex [Standard drug with ClfA **(A)** and ClfB **(B)**] [Candidate molecule with ClfA **(C)** and ClfB **(D)**].

### 3.2 Drug likeness and toxicity analysis of the selected compounds

The drug likeness and toxicity of the selected compound 2-[trans-(4-aminocyclohexyl) amino]-6-(benzyl-amino)-9-cyclopentylpurine] were determined based on the outcome of various standard ADMET parameters. ADMET signifies the absorption of the drug, distribution of the drug, metabolism of the drug, excretion of the drug and toxicity of the drug. The important details of the ADMET properties of the test compounds are summarized in **Tables 2**, **3**.

**Table 2:** Drug-likeness properties of the test compounds compared to those of standards.

	CONSENSUS log P o/w	TPSA (Å^2^)	GI absorption	Violations	Lipinski ‘s test
Molecule1 (Compound)	3.09	93.68	High	0	Passed
Molecule 2 (Standard)	0.20	164.12	Low	3	Passed

**Table 3:** Drug toxicity status of the test compound.

Compound	Hepatotoxicity	Carcinogenesity	Mutagenecity	Cytotoxicity
cdk2_X-ray_inh5_1g5s_1_11	Inactive	Inactive	Inactive	Inactive

### 3.3 Molecular simulation analysis of adhesin ligand interactions

The interactions of the docking complex of the shortlisted compound and standard with ClfA and ClfB were further studied by molecular dynamics simulation.

The root mean square deviation was determined for the interaction of the compound as well as the standard with ClfA and ClfB. The average RMSD of the standard drug allantodapsone for binding ClfA was 1.76 Å, and that for binding to ClfB was 1.17 Å. The average RMSD of the candidate compound/ligand was 1.66 Å and 1.16 Å for the interaction of the standard drug allantodapsone with ClfA and ClfB, respectively (**Figure 2**). An RMSD less than 3 Å is considered a good interaction.

**Figure 3:**
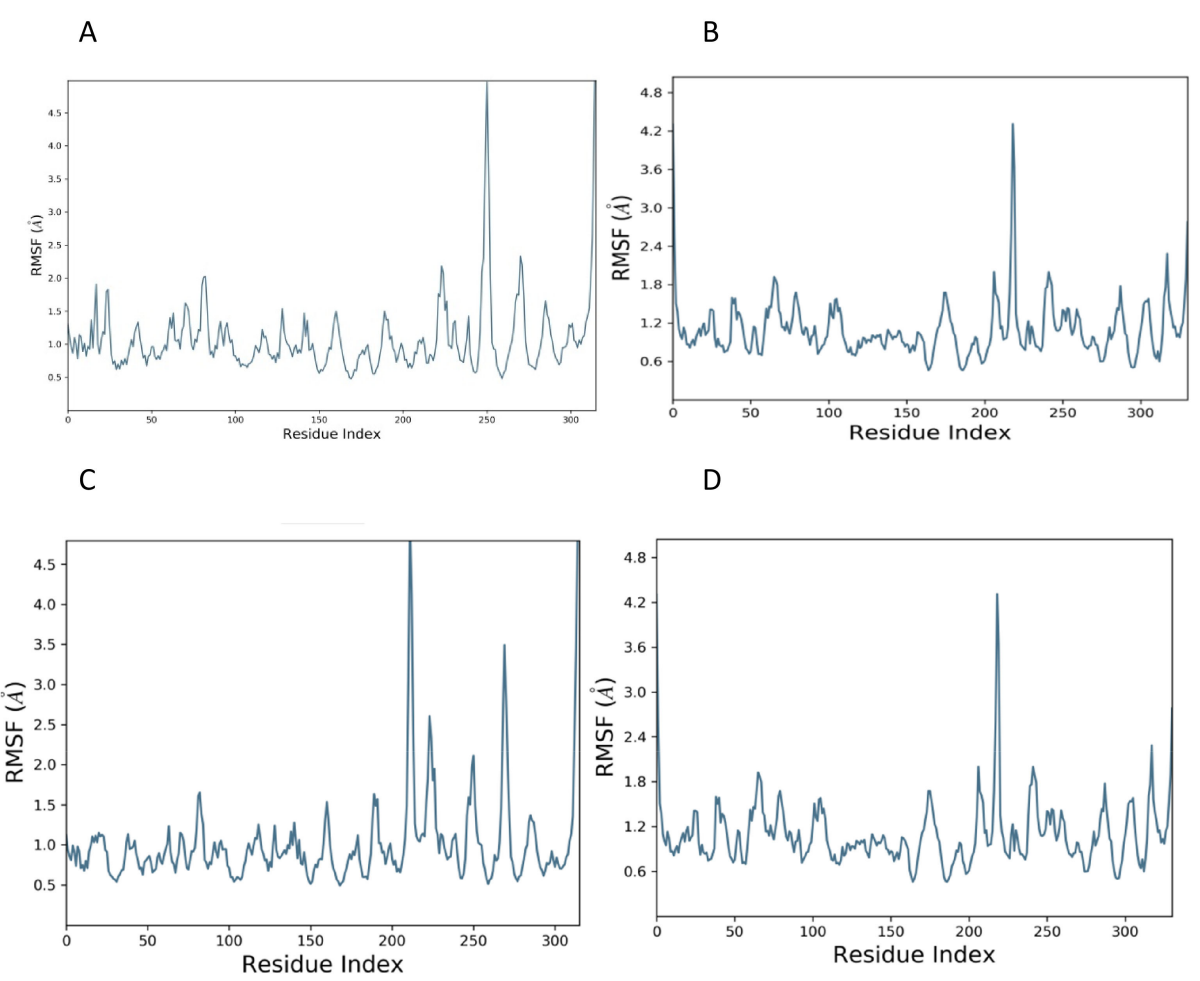
RMSF plot of docked complex [Standard drug with ClfA **(A)** and ClfB **(B)**]; [Candidate molecule with ClfA **(C)** and ClfB **(D)**.

The root mean square fluctuation is a measure of residual fluctuation in a comparative study. The standard showed a peak at the 250^th^ residue in the case of ClfA, while the candidate compound dissipated in areas closer to the 250^th^ residue. In the case of ClfB, the standard as well as the candidate compound showed a peak closer to the 210^th^ residue (**Figure 3**).

Protein–ligand interactions include four types of bonds: ionic, hydrophobic, waterbridge and hydrogen bonds. The majority of the interactions were waterbridge interactions. There were fewer ionic and hydrophobic interactions. Hydrogen bonds were detected between PRO, ASP and GLU in ClfA and between PRO, HIS, GLN, GLU, ASP and ASN in ClfB.

## 4 Discussion

Among the traditional and rational methods of drug design, the latter is known to be more cost effective and efficient. It is also known to be based on the principle of reverse pharmacology, where an efficient protein target is identified and then molecules from a library are selected based on their interaction with the protein (Swinney and Anthony, 2011). In our study, we accomplished structure-based drug design by adopting molecular docking and molecular dynamics, which are the most common and reliable methods used. These methods help in understanding ligand–protein interactions, conformational changes, binding energy, etc (Kalyaanamoorthy and Chen, 2011). As a result of growing antibiotic resistance among *Staphylococcus aureus* due to overuse or misuse, there is an urgent need for the improvement of therapies for treating and controlling infection (Liu et al., 2020; Qureshi et al., 2023). Currently available antibacterial agents for MRSA include vancomycin, linezolid, clindamycin,daptomycin, dalbavancin, oritavancin, telavancin, etc; unfortunately, resistance to these drugs has already been reported worldwide. These drugs may no longer be reliable options for treating MRSA infections. Given these facts, the World Health Organization has included MRSA in the list of six high-priority pathogens that adversely affect public health (Willyard, 2017).

In recent years, many studies in which structure-based drug design has been attempted for the discovery of newer drugs to treat MRSA or staphylococcal infections have been reported. Ye et al., 2022, identified a compound that blocks the transcription of bacterial DNA. The compound worked by inhibiting transcription factors (Ye et al., 2022). Liu et al., 2020, targeted PVL and α-toxin with a compound named *n*-tetradecylphosphocholine (C_14_PC), which inhibits the binding of these virulence toxins to the cell through membrane phosphatidylcholine (Liu et al., 2020). Wang et al., 2018, reported a Sortase B (SrtB) inhibitor, Coptisine, a natural compound that does not exhibit antibacterial activity but can inhibit SrtB activity *in vitro*. In India, Morris et al., 2024, targeted amidase activity by designing the inhibitors SPECS-1 and SPECS-2, which reduced the biofilm-forming activity of bacteria (Morris et al., 2024). Zeenat et al., 2023, reported that the phytochemicals present in curcumin and eugenol are inhibitors of Eap (extracellular protein) homologs 1 and 2. It was tested and validated by molecular docking and dynamics (Zeenat et al., 2023). Rahman & Das (2021) concluded that surface adhesins are very promising as targets for designing vaccines. They identified 53 drug targets that are eligible for vaccine or drug development (Rahman and Das, 2021). Saha & Ghosh, 2023, targeted AgrA, a virulence factor regulator, to develop ligands that inhibit the binding of AgrA to its promoter site (Saha and Ghosh, 2023). Vijayakumar et al., 2022, tested the antibiofilm-forming ability of hesperidin, which is found in citrus fruits. They performed molecular docking to determine the interaction of hesperidin with the biofilm-forming SarA and CrtM proteins (Vijayakumar et al., 2022). Singh et al., 2022, targeted the protein and enzyme saDHFR, which is an important factor for the survival of bacteria (Singh et al., 2022). Sharma et al., 2024, identified inhibitors of crtM and sarA compounds from essential oils. crtM and sarA are proteins involved in biofilm formation (Sharma et al., 2024).

Riaz et al., 2023, in Pakistan, tested the binding affinity of three derivatives of quinolones, namely, sulfanilamide, 4-aminobenzoic acid, and sulfanilic acid, for five bacterial proteins. They found 2 out of 3 derivatives tested to be inhibitors of MRSA (Riaz et al., 2023). Prencipe et al., 2022, also developed the ClfA and B inhibitor allantodapsone, which has been found to have anti-colonizing properties *in vitro* (Prencipe et al., 2022). The present study also investigated the same adhesin proteins, ClfA and ClfB, to develop a ligand compound that interferes with the binding of ClfA and B to the host surface. We used allantodapsone as a standard in our study to validate and compare the findings. By comparison of various parameters, we found that the binding energies of the identified compounds with ClfA and ClfB (-10.37 and -11.11 kcal/mol, respectively) were greater than those of allantodapsone with ClfA and ClfB (-9.25 and -9.09 kcal/mol, respectively). The molecular dynamics results revealed that the interaction of the identified compound with ClfA and ClfB (1.66 Å and 1.16 Å, respectively) was greater than that of the control.

*In silico* identification of ligands and compounds that inhibit bacterial pathogenesis is an advantage for healthcare systems. Because this compound will target and avoid the colonization, an infection will not be established and development of resistance by the bacteria will not take place. Most of the studies that have been conducted on this topic have not been performed at any preclinical or clinical stage. The findings of *in silico* compatibility or interactions provide a foundation for *in vitro* and *in vivo* interactions with actual pathogens. The identified compound must show affinity for its target both *in vitro* and *in vivo* during the preclinical and clinical testing stages (Vijayalakshmi et al., 2014; Riaz et al., 2023). Prencipe et al. concluded that allantodapsone was capable of inhibiting the binding of bacteria to fibrinogen, loricrin and Ck10 components. Due to the shared ligand fibrinogen, allantodapsone was also able to block the interaction of FnBPA with fibrinogen. However, platelet aggregation and agglutination of *S. aureus* were not inhibited in the case of soluble fibrinogen (Prencipe et al., 2022). As mentioned earlier, our study also included ClfA and ClfB adhesins to identify the selected compound. Based on the findings of molecular docking and dynamics, the identified compound is an ideal inhibitor of Clf A and Clf B. Further *in vitro* and *in vivo* studies will confirm its role as a promising drug to prevent colonization and infections.

## 5 Conclusion

*Staphylococcus aureus* is an important pathogen that is currently responsible for the high mortality rate in hospitals and in communities. Its pathogenicity is not only limited to humans but also widely observed in animals. MRSA is the most common resistant form of this organism. Due to increasing resistance to existing antibiotics, treatment has become a challenge, especially in patients with comorbidities or in the ICU. Designing a drug based on the target protein structure/active site and predetermined interaction and its dynamics can help in formulating a drug that is long lasting and promising.

## Data Availability

The original contributions presented in the study are included in the article/supplementary material. Further inquiries can be directed to the corresponding author.
